# Greater cortical thinning and microstructural integrity loss in myotonic dystrophy type 1 compared to myotonic dystrophy type 2

**DOI:** 10.1007/s00415-024-12511-0

**Published:** 2024-06-19

**Authors:** Britta Krieger, Christiane Schneider-Gold, Erhan Genç, Onur Güntürkün, Christian Prehn, Barbara Bellenberg, Carsten Lukas

**Affiliations:** 1 Institute for Neuroradiology, St. Josef Hospital, Ruhr-University-Bochum, Gudrunstr. 56, 44791 Bochum, Germany; 2 Department of Neurology, St. Josef Hospital, Ruhr-University Bochum, Gudrunstr. 56, 44791 Bochum, Germany; 3 Department of Psychology and Neurosciences, Leibniz Research Centre for Working Environment and Human Factors (IfADo), Ardeystraße 67, 44139 Dortmund, Germany; 4 Department of Biopsychology, Institute of Cognitive Neuroscience, Faculty of Psychology, Ruhr University Bochum, 44780 Bochum, Germany

**Keywords:** Myotonic dystrophy, Brain MRI, Cortical thickness, White matter

## Abstract

**Background:**

Myotonic dystrophy is a multisystem disorder characterized by widespread organic involvement including central nervous system symptoms. Although myotonic dystrophy disease types 1 (DM1) and 2 (DM2) cover a similar spectrum of symptoms, more pronounced clinical and brain alterations have been described in DM1. Here, we investigated brain volumetric and white matter alterations in both disease types and compared to healthy controls (HC).

**Methods:**

MRI scans were obtained from 29 DM1, 27 DM2, and 56 HC. We assessed macro- and microstructural brain changes by surface-based analysis of cortical thickness of anatomical images and tract-based spatial statistics of fractional anisotropy (FA) obtained by diffusion-weighted imaging, respectively. Global MRI measures were related to clinical and neuropsychological scores to evaluate their clinical relevance.

**Results:**

Cortical thickness was reduced in both patient groups compared to HC, showing similar patterns of regional distribution in DM1 and DM2 (occipital, temporal, frontal) but more pronounced cortical thinning for DM1. Similarly, FA values showed a widespread decrease in DM1 and DM2 compared to HC. Interestingly, FA was significantly lower in DM1 compared to DM2 within most parts of the brain.

**Conclusion:**

Comparisons between DM1 and DM2 indicate a more pronounced cortical thinning of grey matter and a widespread reduction in microstructural integrity of white matter in DM1. Future studies are required to unravel the underlying and separating mechanisms for the disease courses of the two types and their neuropsychological symptoms.

**Supplementary Information:**

The online version contains supplementary material available at 10.1007/s00415-024-12511-0.

## Introduction

Myotonic dystrophy type 1 (DM1) and 2 (DM2) are dominantly inherited disorders with multisystemic manifestations including not only myotonia and muscles weakness, but also CNS involvement. With a global prevalence of 9.99 cases in 100,000 people worldwide, pooled for both types, it is the most common adult form of muscular dystrophy in adults [1]. The disease course is usually milder in DM2 compared to DM1 [2, 3]. In contrast to DM1, DM2 primarily affects proximal muscles rather than facial or distal muscles. Both forms result from a repeat expansion mutation: a CTG repeat expansions in the dystrophia myotonica-protein kinase gene (DMPK) in DM1 and a CCTG repeat expansion in the zinc finger protein 9 gene (ZNF9) in DM2 [4, 5]. In contrast to DM2 which shows later age at onset, DM1 can occur in each age and is differentiated regarding the time of symptom onset with different CTG repeat lengths: congenital (<1 month, CTG > 1500), infantile (<10 years, CTG 500–1000), classic/juvenile (11–20 years, CTG 400 to >1000), adult (21–40 years, CTG 200–750), and mild late-onset (>41 years, CTG 100–400) DM1 [6]. While myotonia and muscle weakness dominate in adulthood, motoric and mental retardation predominates in the beginning of congenital and infantile forms of DM1 [7, 8].

In recent years, brain involvement in myotonic dystrophy has extensively been studied in particular by MRI. Several studies identified a relationship between cognitive impairment or other clinical symptoms and brain volumetric changes as well as white matter alterations [9–15]. However, their results are partly inconsistent: Daytime sleepiness or visuospatial impairment was significantly correlated with volumes loss in some (sub-)cortical GM structures in one study [10], but another study found only correlations between flexibility of thinking and GM volumes in DM1 (medio-parietal cortex) and DM2 (periaqueductal GM, midbrain, thalamus, parahippocampal gyrus, anterior cingulate) [9]. Further, in contrast to those two studies, a correlation between hippocampal atrophy and nonverbal episodic memory deficits were found in another study [16].

So far, much more MRI studies focused on DM1 than on DM2, which were reviewed by Minnerop et al. in 2018 [17]. As stated in their review, studies analyzing neuroimaging and clinical parameters are inconsistent and not well reproducible across studies, especially for DM2. Thus, previous studies found not only cortical GM reduction, but also subcortical GM reduction [16, 18], whereas others could not detect any GM decrease in DM2 patients [19]. Predominant WM alterations in DM2 affecting the corpus callosum, the cerebellum, and every brain lobes were found by some studies [18, 19], whereas on the contrary, another study revealed a more pronounced GM loss compared to WM atrophy, which was found in the cingulate and in the medial frontal and primary somatosensory cortices [9]. A recent review by Peric et al. (2021) summarized recent progresses and findings of cerebral involvement in DM2 [20]. They highlighted the life restricting cognitive and clinical symptoms of DM2, such as memory and visuospatial impairment, fatigue, and pain.

Only few cross-sectional MRI studies, investigating brain volumetric and diffusion-based alterations, included both disease types [9, 13, 16, 19, 21]. They investigated only small sample sizes of less than 20 patients in most cases, though Minnerop et al. [19] included 22 patients in each group. Although the low prevalence of myotonic dystrophies makes it difficult to collect large cohorts, larger in-depth studies are necessary to further evaluate the role of CNS involvement. In one of those few studies including both DM1 and DM2, both groups showed GM and WM atrophy and enlarged ventricles with a more pronounced motor system involvement in DM1 and a more pronounced affection of the limbic system in DM2 [9]. However, no direct comparison of GM or WM volumes between DM1 and DM2 were described [9]. In another study, in which iron accumulation was investigated by use of quantitative MRI, basal ganglia and DGM nuclei have been shown to have increased iron accumulation in DM1 and DM2, which was more widespread in DM1 [13]. Direct comparison between both disease types yielded higher magnetic susceptibility in the thalamus in DM1 than in DM2 [13].

A comprehensive analysis of brain structure alterations between both groups and the comparison to healthy controls may help to identify differences in affected brain structures and networks. This may help to elucidate differences in severity and patterns of neuropsychological symptoms in DM1 versus DM2 and versus HC. Therefore, our study aimed at identifying macro- and microstructural brain changes regarding both cortical thinning and white matter alterations in a large group of DM1 and DM2 patients. So far, the focus of research on brain alterations has rarely centered on DM2 patients. The extent of structural brain changes with regard to white and grey matter abnormalities described in previous studies revealed to be more pronounced in DM1 [18–20]. Distinct patterns of brain regions involved in DM type 1 and 2 might be related to differences in neuropsychological findings. However, the exact pathophysiological reasons for different patterns of brain regions involved in DM remain to be clarified. Moreover, analysis of cortical thinning patterns and their comparison between both disease types has not been conducted yet, which would give comprehensive and complementary insights into structural abnormalities of DM. Since the mostly used VBM analyses address different structural properties of GM including cortical thickness, surface area, and cortical folding [22], focusing on cortical thickness might reveal more specificity providing complementary information to those from VBM analyses. By comparing patterns of both cortical thinning and white matter alterations between HC, DM1, and DM2, we investigate possible corresponding or complementary structural abnormalities of both measures. Since a higher occurrence of white matter abnormalities than cortical abnormalities has already been described for DM1 [23], we aim at comparing these to DM2 in order to further define the emphases of the two diseases.

## Methods

### Participants

A total of 29 DM1, 29 DM2, and 56 HC were recruited in this study. Two DM2 patients did not receive or discontinued MRI. 24 patients and 13 HC were recruited prospectively between 2020 and 2022, whereas the remaining data were collected from an existing database used for previous studies between 2012 and 2015 [9, 13]. Images were acquired at the same scanner and MRI protocol as with the prospectively acquired participants. Patients older than 18 years were recruited in the neurological clinic if the diagnosis of DM1 or DM2 was molecular-genetically proven. Exclusion criteria were strong dementing symptoms, contraindication for MRI acquisitions, taking psychopharmaca, heart pacemaker, renal insufficiency, pre-existing brain disorders, drug abuse, or severe psychological disorders. DM1 patients were included with both adult (*n* = 24, 83%) and childhood (disease onset at age < 10 years; *n* = 5, 17%) forms. 

### Magnetic resonance imaging

All MRI scans were acquired on a single 3 T Philips Achieva scanner with a 32-channel phased-array head coil. The MRI protocol included a high resolution 3D T1-weighted (T1w) sequence (voxel size: 1 × 1 × 1 mm^3^, field of view: 240 × 240 × 180 mm^3^, TE: 4.6 ms, TR: 10 ms, flip angle: 8°, turbo factor: 164, acquisition time: 6 min), a 3D Fluid attenuated inversion recovery sequence (FLAIR, voxel size: 1 × 1 × 1 mm^3^, field of view: 240 × 240 × 170 mm^3^, TE: 286 ms, TR: 4800 ms, TI: 1650 ms, turbo factor: 182, acquisition time: 6.5 min), and diffusion-weighted imaging (DWI, voxel size: 2.5 × 2.5 × 2.5 mm^3^, field of view: 320 × 240 × 125 mm^3^, TE = 90 ms, TR = 7000 ms, 32 directions, b = 900 s/mm2, phase encoding direction = PA) with one additional b0 image.

### MRI processing

White matter lesion segmentation was conducted using the LST-LPA algorithm in SPM (lesion segmentation toolbox) with FLAIR data and T1w as reference images [24]. Lesion filling was performed to prepare structural T1w images for further analyses. The CAT12 toolbox (version r1932 from 2022–01-13) [25] in SPM was then used to segment brain images and to obtain surface-based parameters including cortical thickness. For that purpose, default parameters were applied.

DTI data were pre-processed using MRtrix functions, which are mostly based on FSL pipelines [26]. These steps included denoising and removal of Gibb’s ringing artifacts. Since no additional acquisitions with reverse phase-encoding direction were performed, distortion correction was conducted using the recently developed approach synb0-disco, which generates an undistorted non-diffusion weighted image from the structural T1w scan based on a deep learning approach [27]. Together with the real b0 image, the synthetic b0 image is then used as a target for distortion correction. The merged b0 images are then input to FSL’s topup function. Conventional eddy current and motion correction was then performed using FSL’s eddy. Diffusion tensor fitting was conducted using FSL’s dtifit to obtain fractional anisotropy (FA), radial diffusivity (RD), and mean diffusivity (MD) maps.

### Clinical examination

All patients underwent a comprehensive neurological examination for clinical symptoms and CNS involvement (CSG). Daytime sleepiness was assessed by scoring according to the Epworth sleepiness scale (ESS). The ESS classifies daytime sleepiness into three stages: 0–10 as “no or moderate sleepiness”, 11–18 as “sleepy” and 19–24 as “very sleepy” [28]. In addition, the presence of a restless-legs syndrome was examined by use of the Cambridge-Hopkins Questionnaire (short version 2) [29]. For DM1, the number of CTG repeats was collected.

Furthermore, the degree of muscular impairment was categorized using the muscular impairment rating score (MIRS), which is a five-point rating scale developed to characterize the distal to proximal progression of muscular involvement in DM1. MIRS was adapted for DM2 patients as described previously [9]. For that, muscular impairment was graded to: grade 1 (no muscular impairment), 2 (minimal signs including myotonia, neck flexor weakness, no proximal weakness), 3 (proximal weakness but no distal weakness except for thumb and deep finger flexor weakness), 4 (proximal and distal weakness), and grade 5 (severe proximal and distal weakness). To overcome potential influences due to adaptation for DM2, the Modified Rankin Scale (MRS) [30] was also calculated as a more general and neutral score for patient’s disability. The MRS measures disability using five scores: 0 (no symptoms), 1 (no significant disability), 2 (slight disability), 3 (moderate disability), 4 (moderately severe disability), 5 (severe disability).

### Neuropsychological testing

Patients were further examined by an experienced neuropsychologist (CP). The standardized neuropsychological test battery included tests for attention, alertness, memory, and executive functions. Specifically, selective attention was assessed by the d2-Test and the Go/no-Go test from the computerized Test for Attentional Performance (TAP). Divided attention and reaction ability (tonic and phasic alertness) were examined by use of TAP. Non-verbal intelligence was evaluated by the intelligence test LPS (Leistungsprüfungssystem) subtest 3 [31], flexibility of thinking by Regensburg verbal fluency test (RWT) [32], the verbal short-term memory by the Wechsler-Memory Scale (WMS–R) with digit span forward and backward [33], non-verbal visual working memory by WMS-R with block tapping forward and backward, verbal memory by Rey auditory verbal learning test (RAVLT), spatial visualization ability by LPS subtest 7, and general intelligence by the clock-drawing test and the Multiple-Choice Vocabulary Intelligence Test (MWT-B). Neuropsychological findings were classified into impaired or unimpaired according to normative data as provided by the specific test manuals. This was done by use of a threshold of one standard deviation below the normative mean. Neuropsychological test results were transformed into *z*-scores to provide scores that are standardized with regard to age, gender, and education. For results of attention tests (d2-test, TAP subtests), that are largely independent from education, the 25–75 percentage range of the normative data was used as expectancy value for evaluation.

In addition, depression was scored using the Beck-Depression-Inventory-II (BDI-II), that graded values of 0–11 as normal, 11–19 as mild, 20–26 as moderate, and >26 as severe depression [34].

### Statistical analysis

Global grey matter (GM), white matter (WM), and cerebrospinal fluid (CSF) volumes, and cortical thickness (CT) obtained from CAT12, total lesion volume (TLV), and global FA were compared between the two patient groups, DM1 and DM2, and HC by use of analysis of covariance (ANCOVA) using R [35]. Age, sex, and TIV (not for TLV, CT, FA) were included as covariates. Results were considered as significant at *p* < 0.01.

To assess local CT alterations, surface-based morphometry (SBM) was performed in CAT12. Again, age and sex were included as covariates. Threshold-free cluster enhancement (TFCE) was applied with 5000 permutations, and FWE correction was used. Results were considered as significant for *p* < 0.01. Brain regions overlapping with significant clusters were printed by the toolbox’s atlas function using the Desikan-Killiany DK40 atlas.

An additional SBM analysis was conducted for the healthy controls regarding their age-related changes of CT to ensure that significant effects were not determined by age-related effects. We did not split the HC group into age-matched groups because this might decrease statistical power as it would result in smaller control groups. Instead, a direct comparison of CT between young and old HC was performed with division at 33 years, resulting in two groups of 28 HC each. Again, TFCE and FWE corrections were applied.

Tract-based spatial statistics (TBSS) was conducted in FSL to evaluate group differences between the two patient groups and healthy controls [36, 37]. The pre-processed FA images were non-linearly registered to the 1 × 1 × 1 mm FMRIB58_FA standard space image. The resulting skeleton image was thresholded at 0.2 to remove the peripheral tract and grey matter [37]. Voxel wise statistics on the skeletonized data were conducted with age and sex as covariates, using FSL’s randomise with TFCE and 5000 permutations. FWE correction was applied to correct for multiple comparisons. Results were considered as significant for *p* < 0.01. Mean global FA values were extracted for each subject by use of fslstats.

Patients and healthy controls might show a different behavior regarding their movement when they are lying in the MRI scanner. Therefore, the individual head movement during DTI acquisition was investigated using the restricted movement RMS file obtained from eddy pipeline. For that, the RMS movement relative to the first volume was averaged over each of the 33 individual images for each subject. As no difference was observed between the three groups (HC: 0.212, DM1: 0.190, DM2: 0.221), the movement did not have to be considered as a potential influencing factor.

Relationships between global MRI parameters, clinical parameters and classifications of pathological or non-pathological neuropsychological scores (see above) were evaluated with Pearson’s correlation analyses using R [35]. Results were considered significant for *p* < 0.05 due to a varying amount of missing neuropsychological scores.

## Results

### Demographic data

Demographic data of the study sample are summarized in Table 1. Patients were randomly included into the study which explains the male–female relation which was therefore disproportional in the DM2 group. All patients were Caucasian patients of different European countries.

**Table 1:** Demographic data of patients and healthy controls

	DM1 *n* = 29	DM2 *n* = 27	HC *n* = 56	*p* value
Age (years)^a^	47 (26, 54)	52 (44, 58)	34 (27, 46)	0.001
Sex^b^				0.200
Female	12 (41%)	18 (67%)	31 (55%)	
Male	17 (59%)	9 (33%)	25 (45%)	
Education^b^				
High school graduation	11 (38%)	3 (11%)		
Advanced technical college	4 (14%)	3 (11%)		
Apprenticeship	1 (3%)	2 (7%)		
Secondary education	4 (14%)	10 (37%)		
General school	4 (14%)	7 (26%)		
Unknown	5 (17%)	2 (7%)		

*p* values were obtained from Kruskal–Wallis rank sum test or Pearson’s Chi-squared test

^a^Median (IQR)

^b^Number (%)

Our healthy controls were younger than both patient groups. Interestingly, DM1 patients were higher educated than DM2 patients, as 38% of DM1 graduated from high school while 37% of DM2 had a secondary school certificate.

### Clinical data

Clinical data for our patient groups are summarized in Table 2. DM1 and DM2 groups did not show differences in disease duration and their ages at disease onset. Restless legs syndrome was present in 8 (30%) of DM2 patients and not in DM1 patients. No differences in muscle score, ESS, nor number of patients with rigor were observed.

**Table 2:** Clinical results for DM1 and DM2 patients

Clinical examination	DM1 *n* = 29	DM2 *n* = 27	*p* value
Disease duration	15 (10, 20)	14 (9, 27)	0.600
Unknown	1	0	
Age at onset	28 (18, 36)	34 (23, 41)	0.200
Unknown	1	0	
Adult/childhood^a^ onset	24/5	NA	
Patients with CNS symptoms	2 (7%)	8 (30%)	**0.017**
Unknown	1	0	
Restless legs syndrome	0 (0%)	8 (30%)	**0.002**
Unknown	1	0	
CTG repeats	380 (262, 750)	NA	
Adult onset	350 (250, 530)		
Childhood onset	900 (750, 920)		
Unknown	2		
Muscle score			
MIRS	2.0 (1.5, 3.0)	2.0 (1.0, 3.0)	0.700
MRS	2.0 (1.0, 3.0)	2.0 (2.0, 2.0)	0.600
Unknown	1	1	
ESS	9.0 (6.5, 12.0)	6.0 (3.0, 11.5)	0.120
Unknown	2	0	

Values are depicted as either median (IQR) or number (%). *p* values were obtained from Wilcoxon rank sum test or Fisher’s exact test

*p* values below 0.05 were highlighted in bold

*ESS* Epworth sleepiness scale; *MIRS* muscular impairment rating score, *MRS* Modified Rankin Scale

^a^Younger than 10

### Neuropsychological results

The numbers of pathological results from neuropsychological tests are summarized in Table 3 for both patient groups. The only test which showed significant differences between DM1 and DM2 was the Block tapping (forward) measuring the non-verbal visual working memory with more pathological results in DM2 than in DM1. A high number of pathological results in more than one third of the patients in DM1 or DM2 was further observed in the flexibility of thinking, which was tested by the verbal fluency test RWT, as well as in verbal short-term memory (WMS-R), spatial visualization (LPS-7, with a trend for higher incidence in DM1), and alertness. While mild depression was frequent in both groups, moderate to severe depression was more present in DM2 patients.

**Table 3:** Neuropsychological test results for DM1 and DM2 patients. Values are depicted as either median (IQR) or number (%)

Neuropsychological scores	DM1 *n* = 29	DM2 *n* = 27	*p* value	Percentile rank (mean (SD))	
				DM1	DM2
BDI					
Points	5 (3, 10)	10 (4, 17)	0.069		
Severity level: mild/moderate to severe depression	5/2	7/5	0.400		
Unknown	1	0			
Selective Attention (d2-test, KL)	5 (22%)	6 (29%)	0.600	58 (31)	57 (32)
Unknown	6	6			
Alertness					
Tonic	10 (37%)	9 (36%)	0.900	37 (24)	39 (30)
Phasic	10 (37%)	12 (48%)	0.400	34 (20)	38 (30)
Unknown	2	2			
Go/no-Go reaction time	6 (23%)	7 (29%)	0.600	56 (31)	55 (31)
Unknown	3	3			
Divided attention, missed events	7 (26%)	10 (40%)	0.300	42 (27)	41 (27)
Unknown	2	2			
Verbal fluency test RWT					
Fluency	7 (26%)	11 (42%)	0.200	48 (23)	42 (30)
Category change	17 (63%)	12 (46%)	0.200	35 (25)	38 (27)
Unknown	2	1			
Digitspan WMS-R					
Numbers forward	7 (26%)	5 (19%)	0.600	58 (31)	62 (32)
Numbers backward	10 (37%)	8 (31%)	0.600	53 (28)	48 (30)
Unknown	2	1			
Block tapping WMS-R					
Forward	9 (33%)	18 (72%)	**0.005**	43 (30)	23 (27)
Backward	13 (48%)	17 (68%)	0.150	38 (30)	33 (26)
Unknown	2	2			
RAVLT					
Course 1	1 (4.2%)	6 (23%)	0.100	80 (14)	66 (28)
Course 5	1 (4.2%)	1 (4.2%)	0.900	90 (22)	82 (25)
Course 6	0 (0%)	1 (4.2%)	0.900	85 (19)	77 (29)
Delay	1 (4.2%)	3 (12%)	0.600	86 (20)	72 (30)
Non-verbal intelligence (LPS 3)	3 (12%)	3 (12%)	0.900	60 (24)	52 (24)
Unknown	5	1			
Spatial Visualization LPS Subtest 7	9 (35%)	4 (15%)	0.110	50 (27)	57 (22)
Unknown	3	1			
Clock test	1 (4.2%)	1 (3.8%)	0.900	1 (0)	1 (0)
Unknown	5	1			
Multiple-Choice Vocabulary Intelligence Test (MWT-B)	3 (12%)	2 (8%)	0.700	56 (24)	64 (26)
Unknown	5	2			

The number of pathological results were summarized. *p* values were obtained from Wilcoxon rank sum test or Fisher’s exact test

*p* values below 0.05 were highlighted in bold

KL: concentration performance value; for the clock test, raw values between 1 (best) and 6 were given instead of percentile ranks

### Lesion distribution

White matter lesions were present in both disease types with similar distributions. Local differences between DM1 and DM2 were merely observed in DM1 showing a higher lesion probability in the bilateral temporal poles, and in DM2 in the right external capsule. Average lesion probability maps for both groups thresholded at 10% are depicted in the supplement (S4).

### Global MRI analysis

**Fig. 1:**
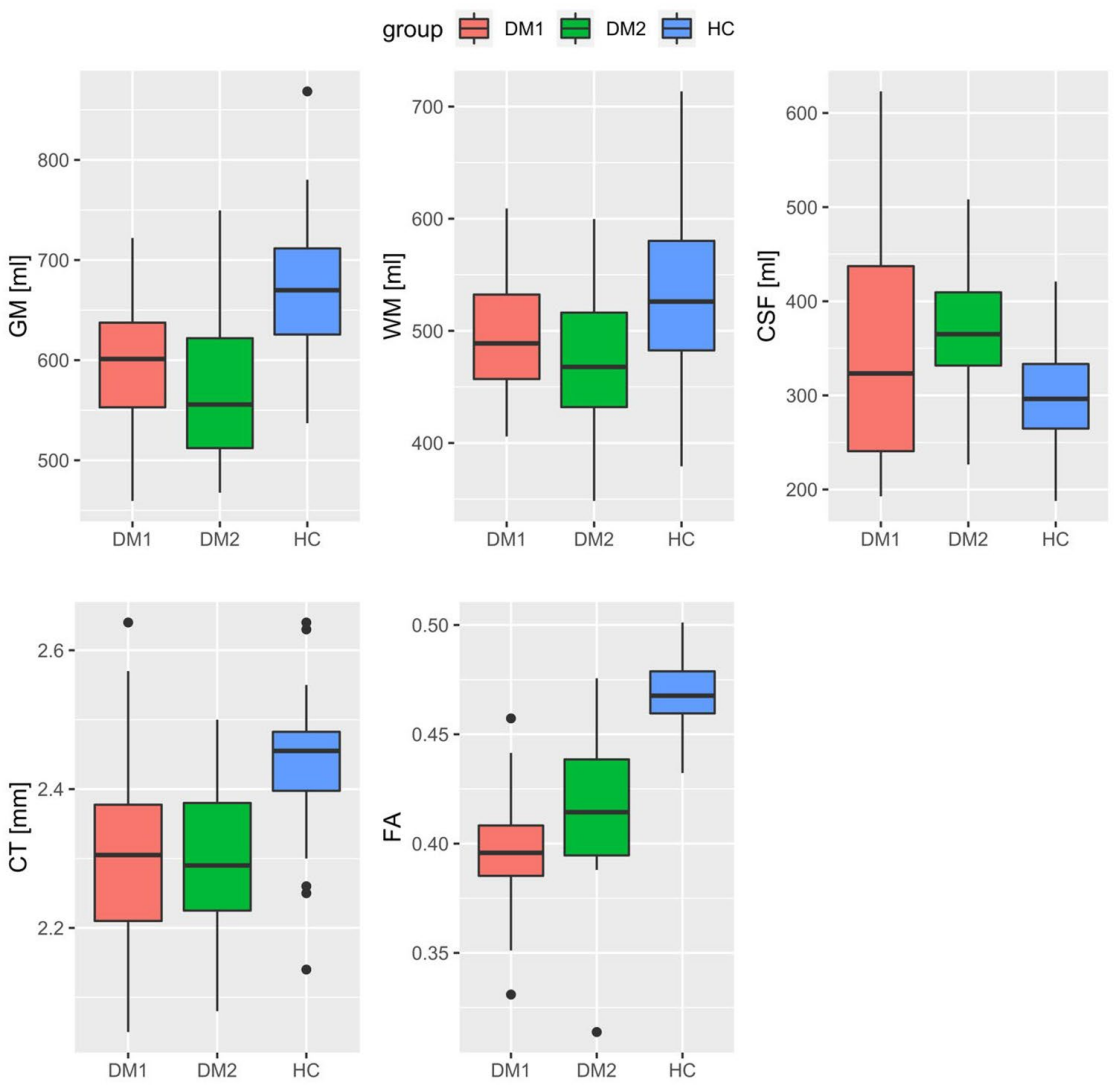
Boxplots of global total gray matter (GM), white matter (WM), and cerebrospinal fluid (CSF), cortical thickness (CT), and fractional anisotropy (FA) for DM1, DM2, and healthy controls (HC)

Across the whole brain, GM and WM volumes and CT were significantly reduced in both DM1 and DM2 patients compared to HC (Fig. 1; Table 4). Furthermore, CSF volumes were increased only in DM2 patients. Similarly, FA was reduced in both patient groups compared to healthy controls. Interestingly, DM1 showed even significantly lower FA values than DM2 (*p* value <0.001, 95% CI [−0.04, −0.01], adjusted difference −0.03).

**Table 4:** Global MRI measures with comparisons between DM1 or DM2 and healthy controls (HC). Values are depicted as mean with standard deviation in parentheses

	HC *n* = 56	DM1 *n* = 29	Adjusted difference^a^	95% CI^a^	*p* value^a^	DM2 *n* = 27	Adjusted difference	95% CI	*p* value
TLV (ml)	0.5 (0.6)	6.8 (9.4)	5.2	2.8, 7.5	**<0.001**	1.4 (16.0)	4.5^a^	−0.29, 9.3^a^	0.065^a^
							2.7^b^	−4.0, 9.4^b^	0.4^b^
GM (ml)	668 (66)	600 (68)	−31	−47, −14	**<0.001**	581 (73)	−32^a^	−46, −18^a^	**<0.001**^a^
							−10^b^	−27, 7.2^b^	0.2^b^
DGM (ml)	33.7 (2.9)	29.9 (3.5)	−2.2	−3.3, −1.1	**<0.001**	29.8 (3.1)	−1.8^a^	−2.7, −0.93^a^	**<0.001**^a^
							−0.93^b^	−2.2, 0.33	0.15^b^
WM (ml)	527 (70)	495 (57)	−5.1	−20, 10	0.500	475 (63)	−22^a^	−35, −9.5^a^	**<0.001**^a^
							14^b^	−4.8, 33^b^	0.14^b^
CSF (ml)	299 (55)	340 (110)	36	10, 61	**0.006**	363 (69)	54^a^	35, 72^a^	**<0.001**^a^
							−4.0^b^	−32, 24^b^	0.8^b^
CT (mm)	2.44 (0.09)	2.31 (0.16)	−0.11	−0.14, −0.07	**<0.001**	2.30 (0.11	−0.07^a^	−0.11, −0.03^a^	**<0.001**^a^
							−0.06^b^	−0.11, −0.01^b^	0.019^b^
FA	0.47 (0.02	0.40 (0.03)	−0.07	−0.08, −0.06	**<0.001**	0.419 (0.032)	−0.04^a^	−0.05, −0.03^a^	**<0.001**^a^
							−0.03^b^	−0.04, −0.01^b^	**<0.001**^b^

*p* values resulted from ANCOVA with age, sex, and TIV (TIV not for TLV, CT, FA) as covariates

*p* values below 0.05 were highlighted in bold

*CI* confidence interval, *TLV* total lesion volume, *GM* gray matter, *DGM* deep gray matter, *WM* white matter, *CSF* cerebrospinal fluid, *CT* cortical thickness, *FA* fractional anisotropy

^a^Compared to healthy controls

^b^Compared to DM1

### Cortical thickness analysis

**Fig. 2:**
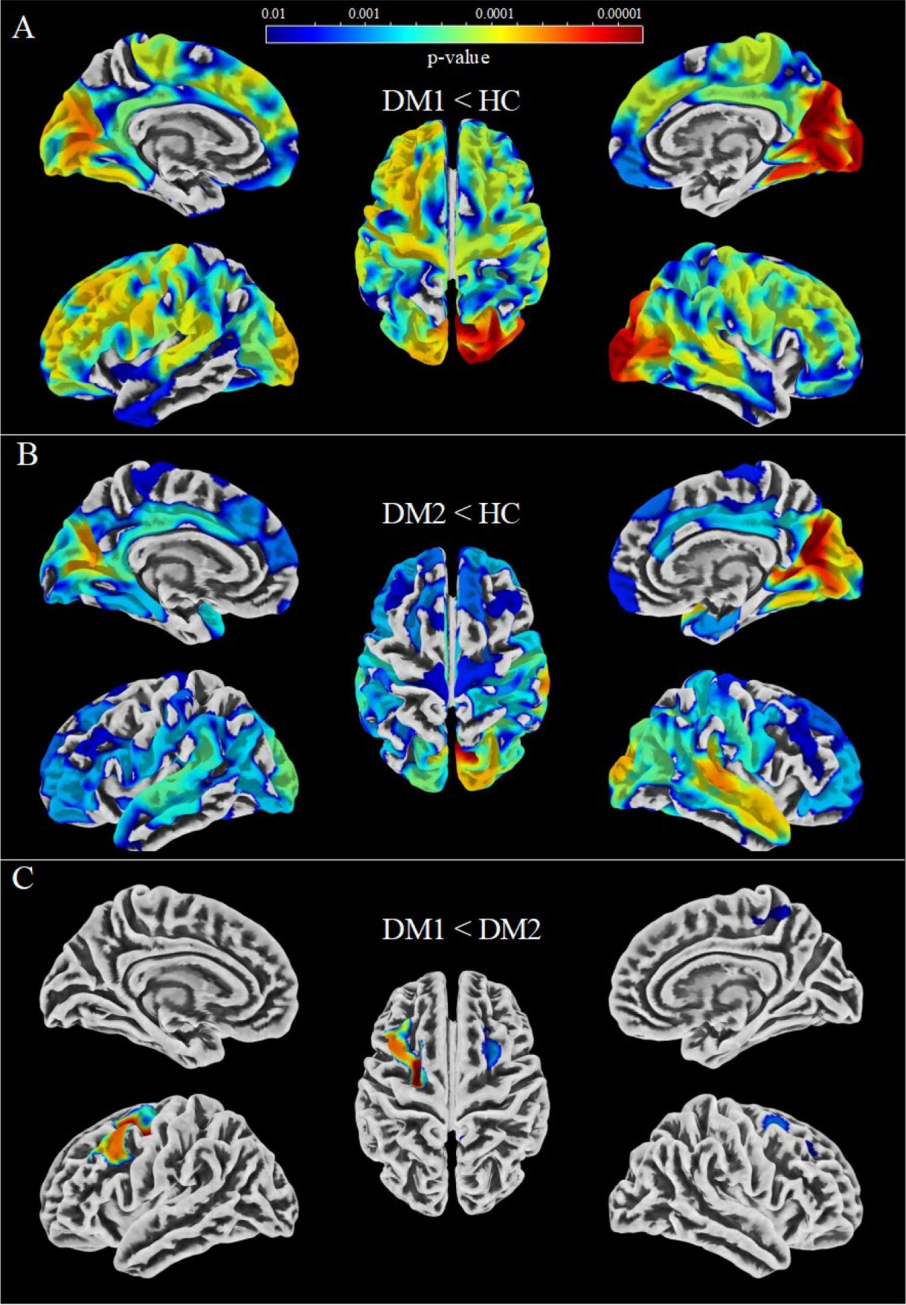
Cortical thickness reduction in DM1 (**A**) and DM2 (**B**) compared to healthy controls, and in DM1 compared to DM2 (**C**). Results were estimated using threshold-free cluster enhancement (TFCE), corrected for multiple comparisons by controlling the family-wise error (FWE), and thresholded at *p* < 0.01

Cortical thinning was present in both disease types, DM1 and DM2, compared to HC in similar brain regions but to a higher extent in DM1 (Fig. 2). Thus, we observed reduced CT in most parts of the occipital lobe in both groups, which was more pronounced in DM1. In both disease types, the right occipital lobe seemed to be more affected than the left lobe. In DM2, the cuneus, precuneus, and lingual gyrus showed most pronounced deviations from HC, whereas in DM1, this pattern was supplemented by the lateral occipital lobe.

Moreover, both groups showed cortical thinning in the superior temporal gyrus, which was more pronounced in DM2 than in DM1. In contrast, DM1 showed a greater extent of CT reduction in the frontal lobe, which covered large parts of the superior, rostral middle, and caudal middle frontal gyrus. Cortical thinning of para-, pre- and postcentral gyri was present in both disease types, though showing stronger effects in DM1.

For the comparison between DM1 and DM2, the caudal middle frontal and parts of the superior frontal and rostral middle frontal lobes showed the most pronounced significant cortical thinning in DM1 compared to DM2. These differences were present in both hemispheres, though much more pronounced in the left hemisphere. This was accompanied by a smaller cluster covering the right precuneus and the right paracentral gyrus. No significant reduction in DM2 compared to DM1 was obtained (data not shown).

A list of atlas regions overlapping with significant cluster is provided in the supplement (S1). As a reference for normal regional cortical thickness distribution, an average image of our HC group is shown in the supplement (S2).

**Fig. 3:**
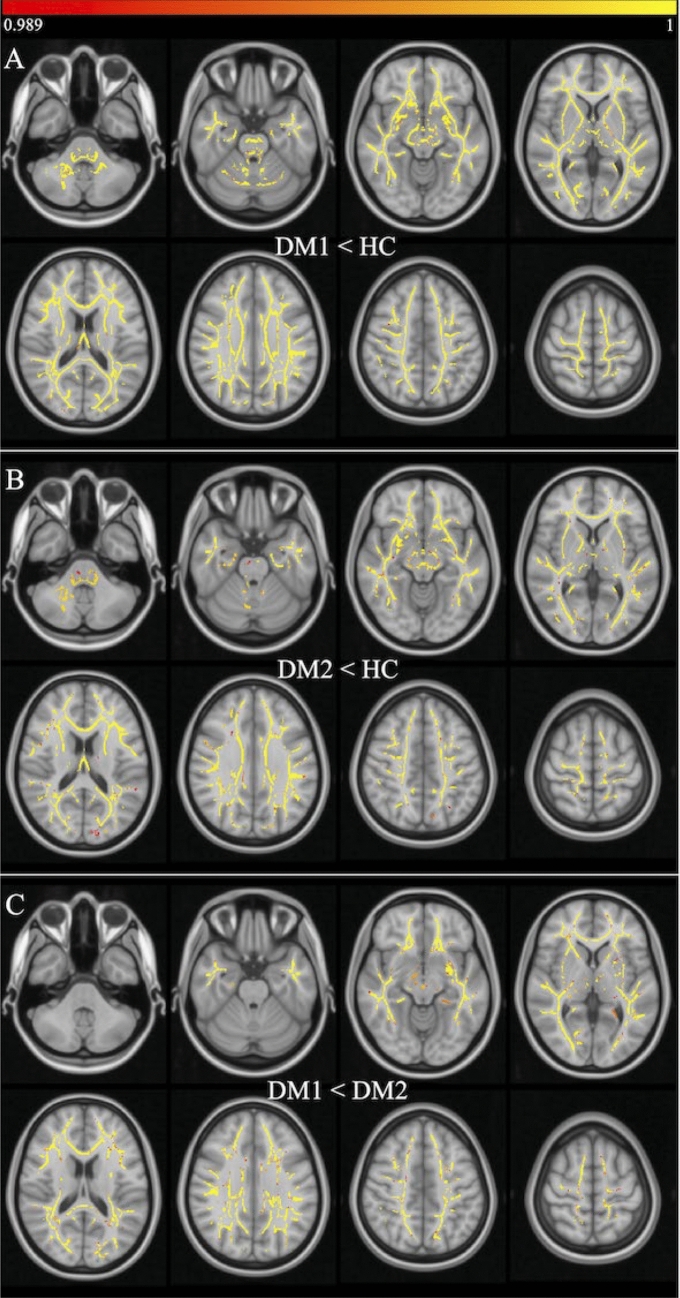
Results from tract-based spatial statistics (TBSS) for comparison of fractional anisotropy (FA) between DM1 and HC (**A**), DM2 and HC (**B**), DM1 and DM2 (**C**). Results were estimated using threshold-free cluster enhancement (TFCE), corrected for multiple comparisons by controlling the family-wise error (FWE), and thresholded at *p* < 0.01. Color scale is shown as values of 1-p

The additional HC analysis between young and older HC yielded smaller CT in most parts of the frontal cortex as well as in the pre- and postcentral gyrus, and parts of the superior and middle temporal gyrus (Supplement Fig. S3). These effects were overall much smaller than the observed CT reductions in the DM groups comparing the ranges of *p* values in Fig. 3 and Fig. S3. This analysis was performed to ensure that our provided disease effects were not mainly driven by age-related effects due to insufficient age-matching of patients and controls. The results showed a strong focus on frontal brain regions and much less occipital involvement, which was obtained for comparison between patients and controls.

### TBSS

Compared to HC, FA was significantly reduced in both DM1 and DM2 across most parts of the white matter skeleton (Fig. 3A, B). Interestingly, we also observed significant decrease of FA in DM1 compared to DM2, which was widely spread across the brain (Fig. 3C). Only few parts of the cerebellum and the pallidum showed no differences. Again, no significant reduction in FA for DM2 compared to DM1 was found (data not shown).

The extent of MD and RD alterations were similarly distributed to FA decrease in DM1 patients, but in DM2, much less regions were affected by MD or RD increases (Supplement S3).

### Correlation between global MRI parameters, clinical and neuropsychological data

Correlation analyses between clinical data and global CT or mean FA yielded a significant negative association between the muscle score and CT in DM1 and DM2, as well as a negative relationship between disease duration and CT in DM2. CTG repeat length was positively related to CT in DM1 patients. FA was negatively associated with disease duration in DM1, for which a trend was also visible for DM2. Results are summarized in Table 5.

**Table 5:** Results from Pearson correlation analyses between cortical thickness or fractional anisotropy (FA) and clinical measures

	Cortical thickness (mm)				FA			
	DM1		DM2		DM1		DM2	
	*r*	*p* value [CI]	*r*	*p* value [CI]	*r*	*p* value [CI]	*r*	*p* value [CI]
Disease duration	−0.29	0.13 [−0.59, 0.09]	**−0.45**	**0.019 [−0.71, −0.08]**	**−0.47**	**0.01 [−0.71, −0.11]**	−0.36	0.08 [−0.67, 0.05]
ESS	−0.34	0.086 [−0.63, 0.05]	0.17	0.39 [−0.22, 0.52]	−0.10	0.62 [−0.46, 0.29]	0.17	0.43 [−0.25, 0.54]
MRS	**−0.48**	**0.01 [−0.72, −0.16]**	**−0.61**	**0.001 [−0.81, −0.3]**	−0.20	0.297 [−0.54, 0.18]	−0.35	0.099 [−0.67, 0.07]
CTG repeat length	**0.53**	**0.005 [0.18, 0.76]**	NA	NA	−0.08	0.68 [−0.45, 0.31]	NA	NA

*p* values below 0.05 were highlighted in bold

ESS Epworth sleepiness scale, *MRS* modified Rankin scale, *CI* confidence interval

For relationships with neuropsychological test results (Table 6), only those tests that were pathological in at least 20% of the patients in each group were considered for analysis to ensure explanatory power. We observed associations between decreased non-visual working memory measured by the block tapping test and decreased CT in both DM1 (backward) and DM2 (forward). An impaired ability of divided attention was further related to decreased FA in DM2. Selective attention assessed by the d2-test was negatively associated with CT in DM1 and with FA in DM2.

**Table 6:** Results from Pearson correlation analyses between cortical thickness or fractional anisotropy (FA) and neuropsychological test results

	Cortical thickness				FA			
	DM1		DM2		DM1		DM2	
	*r*	*p*	*r*	*p*	*r*	*p*	*r*	*p*
Alertness, tonic	−0.17	0.403	−0.05	0.826	−0.30	0.129	−0.02	0.926
Alertness, phasic	−0.24	0.229	0.19	0.357	−0.34	0.079	−0.15	0.515
Block tapping WMS-R, forward	0.04	0.852	**−0.42**	**0.035**	0.08	0.705	0.06	0.785
Block tapping WMS-R, backward	**−0.50**	**0.008**	−0.01	0.955	−0.15	0.457	−0.13	0.559
Divided attention, missed events	−0.26	0.190	−0.09	0.653	−0.18	0.379	**−0.48**	**0.023**
Go/no-Go, reaction time	−0.36	0.070	−0.17	0.417	−0.24	0.235	0.09	0.711
Verbal fluency test RWT, fluency	0.03	0.899	0.29	0.158	0.01	0.976	−0.15	0.487
Verbal fluency test RWT, category change	−0.37	0.057	0.20	0.317	−0.25	0.206	0.06	0.793
WMSR subtest visual	−0.10	0.614	−0.23	0.264	0.04	0.853	−0.39	0.066
d2-test, KL	**−0.52**	**0.011**	−0.1	0.668	−0.34	0.108	**−0.51**	**0.029**
Digitspan WMS-R, backward	0.23	0.257	0.00	0.991	0.04	0.853	−0.37	0.085

*p* values below 0.05 were highlighted in bold

## Discussion

### Cortical thickness

In this study, we provided first evidence for pathological cortical thinning in DM2 patients, which showed mostly overlapping regions but varying extents compared to DM1. Regarding clusters of cortical thinning compared to healthy controls both disease types showed largest cluster extent in occipital lobes accompanied by a stronger focus in the temporal lobes in DM2 and larger alterations in the frontal lobes of DM1 patients.

To our knowledge, this is the first study analyzing cortical thinning in DM2. Regarding cortical thinning in DM1, our results are to a large extent in line with those of comparable studies, although differences in detail may be due to differences in study design or patient selection: a previous study by Serra et al. showed reduced CT in the precuneus, the angular gyrus, the superior temporal gyrus and the medial frontal gyrus bilaterally, the right precentral gyrus, the right posterior and anterior cingulate cortex, and the left superior parietal lobule [38]. Although the anterior part of the cingulate cortex showed no significant differences in our sample, the other named brain regions revealed also decreased thickness. Pronounced thinning was also present in the occipital cortex in our DM1 sample which was not shown to be significant by Serra et al. [38]. However, they found a relationship between CT in the lateral occipital cortex and the performance at the Social Cognition Battery. In addition, another study by Zanigni et al. showed a “prevalent involvement of parietal-occipital region” especially the lateral occipital parts [12], which were highly significant in our study, too. Another study by Yoo et al. showed reduced CT in superior temporal cortices (including the supramarginal cortex), precuneus, lateral occipital cortex, superior frontal, precentral, and postcentral cortices in the patient group compared to controls [23], covering all brain regions that were also significant in our sample.

In our study sample, reduced visuospatial functioning and deficits of the non-verbal visual working memory accompanied occipital cortical thinning. Although visual deficits were not reported, this might be evidence for disturbances of visual processing within the occipital lobes. Further, the temporal lobe was affected by cortical thinning, which was more pronounced in DM2, were patients showed a high number of pathological results for the non-verbal visual working memory test. Especially, the medial temporal lobe plays a pivotal role in visual working memory, which was shown in our patients. Taken together, this provides evidence for disturbances of the occipito-temporal region in DM which has a large impact on processing of visuospatial information through the ventral visual pathway. It has been recently described using tractography analyses that the medial occipital longitudinal tract has a major function for visuospatial learning as it seemed to carry visual information between the early visual cortex and parahippocampal area [39].

Our HC were relatively younger than DM1 patients, but we think that this does not have a major impact on our results. Cortical thinning was more pronounced in DM and even more sever in DM1 despite DM2 patients were even a slightly older than DM1 patients. Based on our additional analysis between young and older HC, we proposed that our shown disease effects were not mainly driven by age-related effects. Thus, we showed that CT in HC who were younger than 33 years was not significantly decreased in occipital brain regions, instead a strong focus on frontal brain regions and post- and precentral gyri was obtained. This might not completely replace a perfectly age-matched control group, but with regard to our amount of available data, this additional analysis should provide more reliability of our results and weaken the influence of age for interpreting our results.

Direct comparison of CT between DM1 and DM2 yielded differences in the frontal lobes, the precuneus and the para- and precentral gyri where CT was lower for DM1 than for DM2. Like other parietal areas, the precuneus is relevant for visuospatial functions [40]. Clinically, our DM1 sample showed more pronounced daytime sleepiness and less spatial visualization abilities compared to DM2, though other parameters were either comparable or more decreased in DM2 such as the non-verbal visual working memory. In a recent study, acute sleep deprivation was shown to be associated with decreased functional connectivity between the precuneus and the middle frontal gyrus in healthy subjects [41], but also other studies analyzing healthy subjects found relationships between frontal lobe metabolic decreases and sleep deprivation [42]. Since these metabolic changes were only partially reversed by recovery sleep, we hypothesized that long-term consequences of cortical thinning might be involved. Thus, our DM1 patients who are suffering from daytime sleepiness, which is often accompanied by a disturbed sleep rhythm, might have subsequently developed CT alterations. Sleep disturbances are also known to be a factor for promoting dementia, which could be avoided by appropriate treatment. Therefore, treating sleep disturbances in DM 1 and DM2 may be a potential therapeutic approach in order to prevent progression in cortical thinning.

### Microstructural white matter alterations

The presented widespread reduction in FA provided evidence for microstructural alterations in both DM1 and DM2. As previously shown, this gives an indication of decreased white matter integrity [43]. As it has been already shown by Minnerop et al. [19], motoric tracts were strongly affected by decreased FA in DM1. In our study, both patient groups showed abnormal neuronal integrity in temporal lobes, which was also more pronounced in DM1. Similarly, cerebellum and brainstem were affected in both groups, but this tended to be less in DM2 again. In general, the more pronounced FA decrease in DM1 could be caused by its genetic effect which might have a larger impact on several distinct cell types in DM1, addressed recently by Lopez-Martinez et al. [44]. As described in their review, alternative splicing misregulations occur in cells of DM1 patients in different tissues such as brain tissue. Missplicing of the NMDA receptor 1 or the microtubule-associated protein tau was described to contribute to memory impairment or tauopathy-like degeneration of brain tissue, respectively. Since tau aggregations have been described to be associated with WM microstructural alterations in Alzheimer’s disease [45, 46], the tau-pathology could be also related to a pronounced FA decrease in DM1 leading to a widespread impact on several brain structures.

Since white matter was proved to be affected beyond white matter T2 lesions, FA findings indicate pathological disturbances of microstructural barriers to be present also in normal appearing white matter. However, the meaning and underlying causes for such microstructural damage remains unclear. In general, we observed similar lesion distributions for both disease types, with temporal white matter lesions seemed to be only occurring in DM1, which has been already shown by Minnerop et al. [19].

Similar to our study, Minnerop et al. (2011) found widespread differences in DTI measures between DM1 and HC, and less between DM2 and HC [19]. Comparing DM1 and DM2, they found less widespread differences between the two groups compared to our study. Although they did not describe the direct comparison between DM1 and DM2 regarding MD and RD changes, the comparison of both groups to HC yielded similar results to those we found. Thus, MD and RD were much more increased in DM1 than in DM2 when compared to HC.

In another investigation of cerebral FA alteration in a small sample of DM1 and DM2, the tendency of lower FA in DM1 compared to DM2 was shown, though only a rough division into larger brain lobes was performed and only five DM2 patients were included [47]. To our knowledge, no other study performed a comparison of white matter alterations between both disease types.

Other studies focused only on DM1 patients. For example, Baldanzi et al. (2016) also found extensive FA decreases compared to HC [48]. In another study investigating tractography, disruptions of white matter integrity were described for all tracts [49].

### Clinical relationship with global MRI metrics

Both groups showed a relationship between disease duration and global MRI measures, but with different focuses. Thus, lower cortical thickness was associated with disease duration in DM2, while DM1 showed a significant relationship between disease duration and global FA decrease. This might be evidence for a different focus of brain alterations in the two disease types. Changes over time should be further analyzed in a longitudinal manner. Only two studies investigated longitudinal brain changes in DM1 [11, 50], and only one study included both types [51]. For example, Labayru et al. [50] found a progressive microstructural WM impairment in DM1, as global FA values were decreased over time.

In addition, a higher grade of muscular impairment was associated with decreased CT in both DM1 and DM2, but not with FA, which gave rise to the assumption that CT might be an imaging marker more related to muscle weakness and disability measured by MRS than global WM microstructural degradation. Still, there might be specific regions connected to muscular impairment rather than global WM integrity, which was not analyzed here. Based on our results, it would be worth to further investigate regional CT in relationship to physical constraints.

Surprisingly, CTG repeat length was positively related to CT in DM1. This contradicts other studies [23] and might be influenced by the inclusion of five childhood-onset patient. Childhood-onset patients showed significantly higher number of CTG repeats than adult-onset patients (Table 2) and simultaneously higher CT values (Supplement S5). Thus, there seemed to be an effect of high CTG repeat lengths on global CT, which had a high impact on the overall trend of the relationship between CTG repeats and CT and overshadowed the assumed negative association. Higher CT in childhood-onset DM1 might be explainable by the assumption that in younger ages, patients are able to compensate for atrophy and therefore they might not develop a rapid cortical thinning over a similar duration of the disease course as adult onset patients. No differences with regard to FA could be obtained for high or low numbers of CTG repeats, so that there might exist a distinguishable impact of repeat lengths on either FA or CT. We included both types of disease onset of DM1 in the other analyses as our childhood-onset patients showed only mild cognitive deficits and had no noticeable findings in their test results.

As far as we know, no differences between both patient groups regarding education have been described elsewhere. In contrast to the assumption that cognitive impairment in DM1 is usually more pronounced in DM1 than in DM2 the educational level of our DM1 patients was higher than in our DM2 patients. On the one hand, this could be related to a selection bias introduced by recruiting patients being interested and willing to participate in a clinical study, but this would account for both groups. On the other hand, our group of DM1 patients was obviously not a negative selection of cognitively impaired patients and therefore the neuropsychological and MRI findings are even more interesting and valuable. The findings indicate that in our DM1 patients, CTG repeat expansion sizes within the range of 120–1000 repeats were not generally associated with lower intelligence, frontal and memory dysfunction or reduced cognitive performance related to daytime sleepiness. This contradicts previous studies in which some neuropsychological test results were correlated to CTG repeat size [52, 53]. This could be related to genetic mosaicism [54] or selection bias towards inclusion of more educated patients into the study. Nevertheless, the educational levels in our DM1 patients and their neuropsychological test results underline that there is a strong need for individual neuropsychological testing to reduce inadequate presumptions and also for the evaluation of educational and professional levels in larger cohorts of DM1 and DM2 patients.

### Neuropsychology

The importance of macro- and microstructural brain changes for cognitive functioning was shown by several associations between global CT or FA and pathologically relevant neuropsychological test results. Thus, complementary relationships were obtained for CT and FA, which were also differing between DM1 and DM2. Impaired selective attention, assessed by the d2-Test, was associated with global cortical thinning in DM1, but not in DM2, though the latter group showed a relationship to decreasing FA. Similarly, divided attention was negatively related to FA in DM2, but not in DM1. Therefore, attention deficits might underlie different mechanisms in the two disease types or have different extents in varying brain regions that might be functionally compensated by other regions. Again, based on our global analyses, it would be worth to further investigate regional CT and FA in relationship to neuropsychological alterations.

Previous studies that investigated the relationship between brain imaging measures and neuropsychology revealed heterogeneous results as reviewed by Minnerop et al. [17]. In one study, atrophy in the visual cortex was associated with reduced flexibility of thinking in DM1 and in DM2, depression was associated with brainstem atrophy and daytime sleepiness was correlated with atrophy in the middle cerebellar peduncles, pons, and the right medio-frontal cortex [9].

Regarding white matter integrity and cognitive decline, another study described a significant correlation between visual memory and WM integrity in the anterior cingulum, the splenium of the corpus callosum, and the precuneus [55]. Those analyses are indeed more specific compared to our global associations, which might be an explanation for our lacking relationship between, for example, selective attention and FA in DM1, and which would further point to the assumption of a compensatory effect by different brain regions. For DM2, results of the few existing studies are contradictory: in one study, no correlations between global brain volume and clinical parameters were found [21], whereas another study revealed significant correlations between brain volume and visuo-constructive abilities or psychomotor speed [16]. The involvement of GM abnormalities for visual abilities were confirmed in our study as cortical thinning was found to be accompanied by reduced visuospatial functioning. However, on one hand, small sample sizes in the mentioned previous studies of nine DM2 patients have to be considered. On the other hand, the comparability of the used neuropsychological tests are questionable.

### Macro- and microstructural brain changes

Brain regions affected by white matter integrity alterations were widespread across the whole brain, so that an overlap with the more distinct cortical thinning was shown accordingly. Thus, microstructural changes seemed to be present beyond CT alterations. This is in line with previous studies suggesting that DM is a predominant white matter disease [12, 19]. On one hand, white matter changes might constitute an additional or underlying pathological mechanism with higher vulnerability. On the other hand, CT might provide a more specific and distinct biomarker for disease activity than DTI measures. Whether white matter changes were progressively increased and whether cortical thinning occurred later or to a lesser extent over time remains unclear and could be analyzed longitudinally. In the few existing longitudinal MRI studies, analyses did not comprise both CT and DTI measures [11, 50, 51], though it would provide evidence for their temporal evolution and their relationships.

### Limitations

Some limitations must be considered for interpretation of our results. First, this study might be based in absolute terms on a small sample of patients, although the number of patients is higher compared to the other studies. DM is a rare disease, which limits the possibilities to recruit patients that correspond to our strict inclusion criteria.

Second, no distortion correction could be done with raw data, because older scans were acquired without additional b0 with opposite phase encoding direction. This method was developed in the past years so that it was not common 8–10 years ago when our first dataset was acquired. Therefore, all data were handled the same and methods were not mixed.

### Conclusion

Cortical thinning in DM is the main finding in our MRI analysis and was shown to be more widespread in DM1 as compared to DM2. There are overlapping pathological mechanisms in the two disease forms as shown by similarities in cortical thinning patterns. However, differences in micro- and macrostructural characteristics were observed as DM1 showed more pronounced deviations in FA and CT from HC. Our results might indicate the involvement of a high number of different neuronal cell types and of a more complex pathology. Cortical thinning in DM2 seemed to have a focus in the temporal lobe in addition to the occipital lobe, which is common in both disease types. These characteristics might help to further differentiate both types.

To sum up, we confirmed previously shown affection of brain white and grey matter in both disease types by use of larger sample size in comparison to previous studies and additionally showed extensive differences in white matter integrity between DM1 and DM2.

### Supplementary Information

Below is the link to the electronic supplementary material.

Supplementary file1 (DOCX 2055 KB)

## Abbreviations

CNS: Central nervous system
CSF: Cerebrospinal fluid
CT: Cortical thickness
DM: Myotonic dystrophy
ESS: Epworth sleepiness scale
FA: Fractional anisotropy
FLAIR: Fluid attenuated inversion recovery sequence
GM: Gray matter
HC: Healthy control
MD: Mean diffusivity
MIRS: Muscular impairment rating score
MRI: Magnetic resonance imaging
MRS: Modified Rankin Scale
RD: Radial diffusivity
SBM: Surface-based morphometry
TBSS: Tract-based spatial statistics
TFCE: Threshold-free cluster enhancement
TLV: Total lesion volume
WM: White matter
