# Supplementary material for: Greater cortical thinning and microstructural integrity loss in myotonic dystrophy type 1 compared to myotonic dystrophy type 2
Source: J Neurol. 2024 Jun 19;271(8):5525–40. doi: 10.1007/s00415-024-12511-0 (PMC11319366; doi:10.1007/s00415-024-12511-0)

**Supplement**

**S1 Cortical thickness analysis: significant cluster**

TFCE, FWE, p < 0.01

DM1 < HC

______________________________________________________

Desikan-Killiany DK40 Atlas: lh

______________________________________________________

P-value Cluster-Size Overlap of atlas region

0.00006 23675 10% superiorfrontal

8% precentral

8% postcentral

7% supramarginal

7% superiorparietal

7% rostralmiddlefrontal

6% inferiorparietal

5% lateraloccipital

4% superiortemporal

4% precuneus

3% caudalmiddlefrontal

3% lingual

3% fusiform

3% paracentral

2% parsopercularis

2% posteriorcingulate

2% isthmuscingulate

2% lateralorbitofrontal

2% pericalcarine

1% cuneus

1% parstriangularis

1% middletemporal

1% insula

1% bankssts

1% medialorbitofrontal

______________________________________________________

Desikan-Killiany DK40 Atlas: rh

____________________________________________________

P-value Cluster-Size Overlap of atlas region

0.00002 24395 9% superiorfrontal

8% precentral

8% superiorparietal

7% inferiorparietal

7% postcentral

7% supramarginal

6% rostralmiddlefrontal

6% precuneus

4% lateraloccipital

4% middletemporal

3% superiortemporal

3% lingual

3% paracentral

3% caudalmiddlefrontal

3% posteriorcingulate

2% fusiform

2% lateralorbitofrontal

2% bankssts

2% parsopercularis

1% pericalcarine

1% isthmuscingulate

1% cuneus

1% parstriangularis

1% medialorbitofrontal

DM2 < HC

______________________________________________________

Desikan-Killiany DK40 Atlas: lh

______________________________________________________

P-value Cluster-Size Overlap of atlas region

0.00004 18438 8% superiorfrontal

7% supramarginal

7% superiortemporal

7% rostralmiddlefrontal

6% postcentral

6% superiorparietal

6% lateraloccipital

5% precentral

5% inferiorparietal

5% precuneus

4% lingual

3% middletemporal

3% parsopercularis

3% posteriorcingulate

2% fusiform

2% isthmuscingulate

2% parstriangularis

2% paracentral

2% cuneus

2% bankssts

2% insula

2% pericalcarine

2% parahippocampal

1% caudalmiddlefrontal

1% caudalanteriorcingulate

1% lateralorbitofrontal

1% transversetemporal

______________________________________________________

Desikan-Killiany DK40 Atlas: rh

______________________________________________________

P-value Cluster-Size Overlap of atlas region

0.00001 20353 9% inferiorparietal

8% postcentral

8% superiorparietal

8% precentral

7% supramarginal

6% superiortemporal

6% superiorfrontal

5% rostralmiddlefrontal

5% precuneus

4% lateraloccipital

4% middletemporal

4% lingual

3% fusiform

3% posteriorcingulate

2% paracentral

2% bankssts

2% pericalcarine

2% cuneus

1% caudalmiddlefrontal

1% isthmuscingulate

1% lateralorbitofrontal

1% parstriangularis

1% parsorbitalis

DM1 < DM2

______________________________________________________

Desikan-Killiany DK40 Atlas: lh

______________________________________________________

P-value Cluster-Size Overlap of atlas region

0.00765 881 50% caudalmiddlefrontal

28% superiorfrontal

11% rostralmiddlefrontal

10% precentral

______________________________________________________

Desikan-Killiany DK40 Atlas: rh

______________________________________________________

P-value Cluster-Size Overlap of atlas region

0.00925 377 64% superiorfrontal

34% caudalmiddlefrontal

2% rostralmiddlefrontal

0.00981 149 60% precuneus

40% paracentral

**S2 Average cortical thickness (in mm) of healthy controls.**


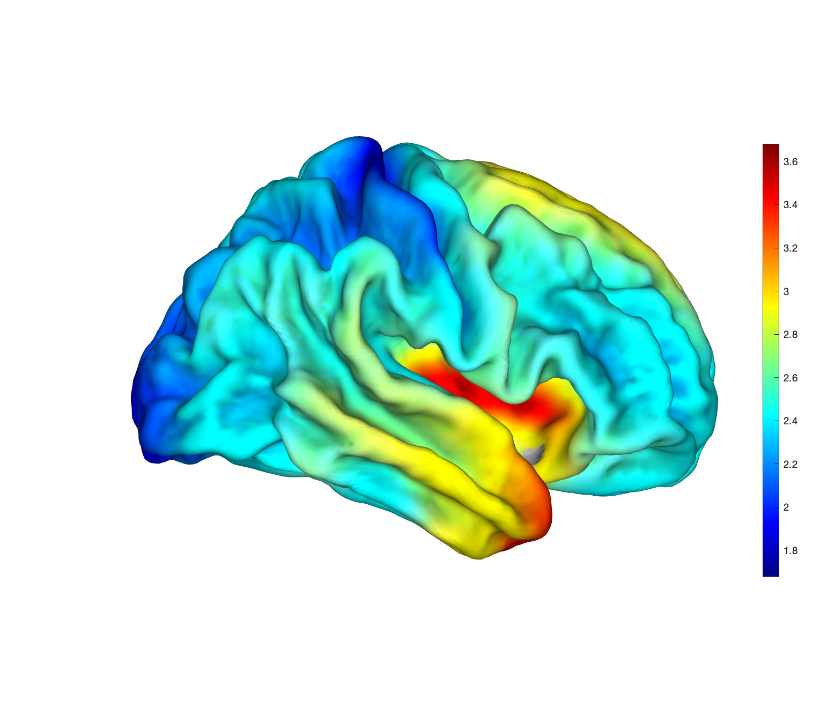


**S3 Cortical thickness analysis for comparison between young (<33y) and old (>33y) healthy controls. Results were corrected by TFCE and FWE and thresholded at p<0.01 .**


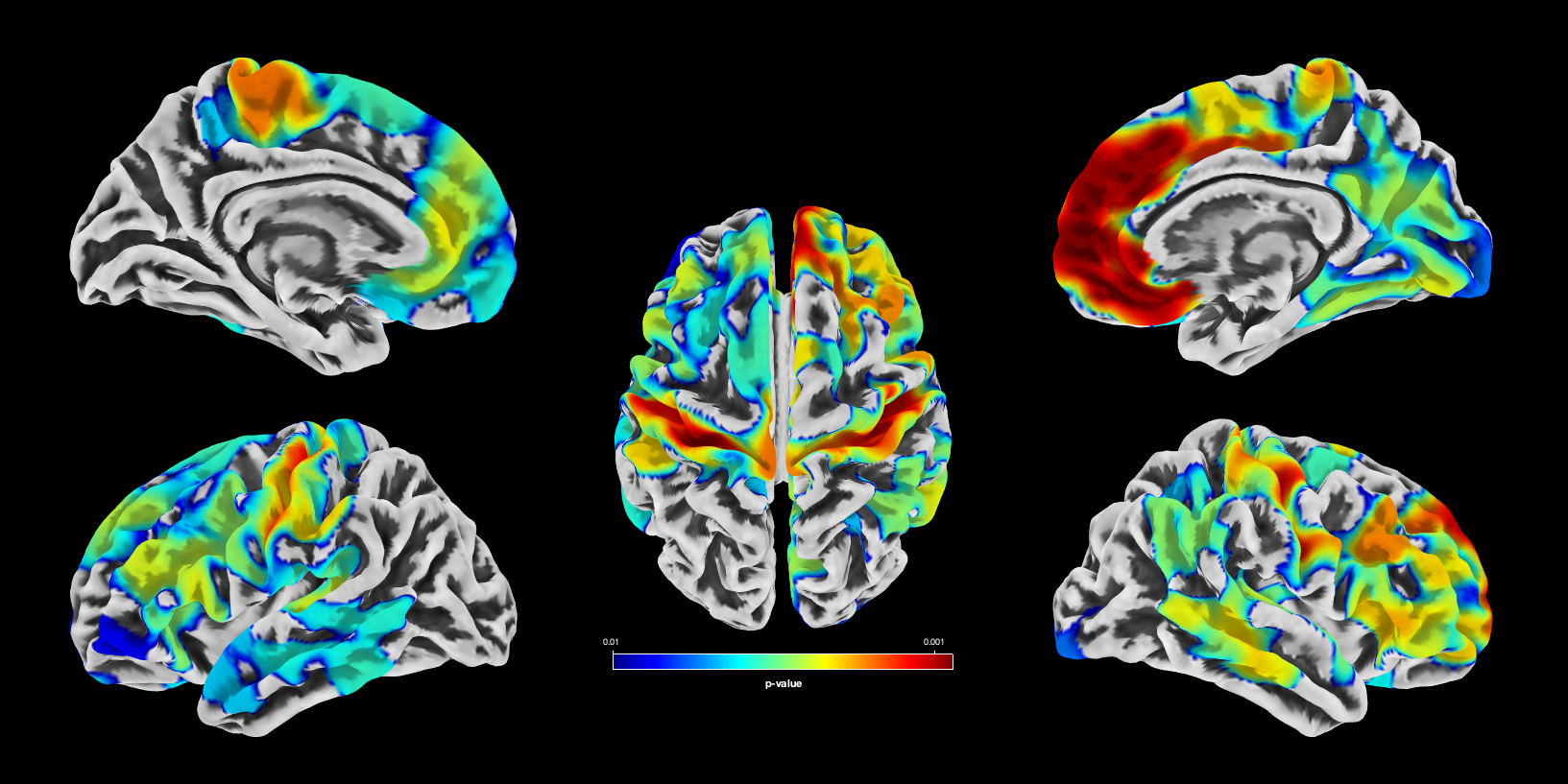


**S4 TBSS**

MD (red), RD (yellow) DM1 > HC


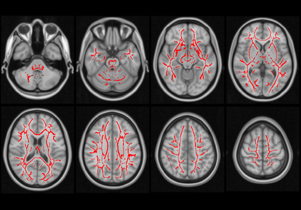

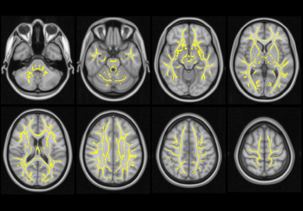


MD (red), RD (yellow) DM2 > HC


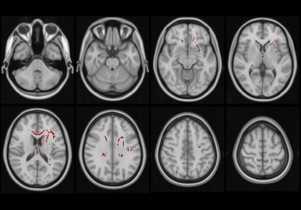

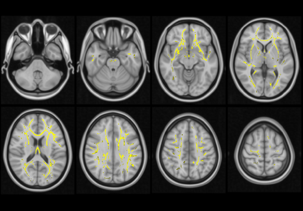


MD (red), RD (yellow), DM1 > DM2


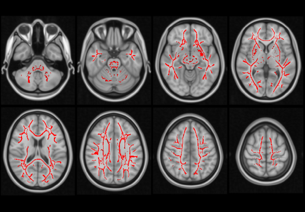

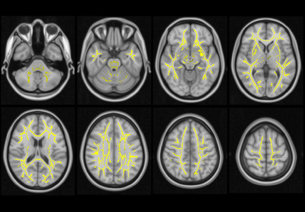


**S5 Lesion distribution: (left: DM1, right: DM2)**


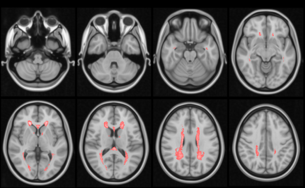

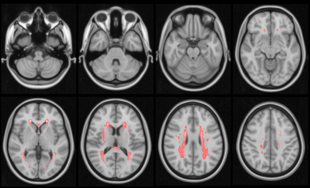


**S6 Comparison of cortical thickness between childhood and adult onset DM1 patients with regard to CTG repeat length**


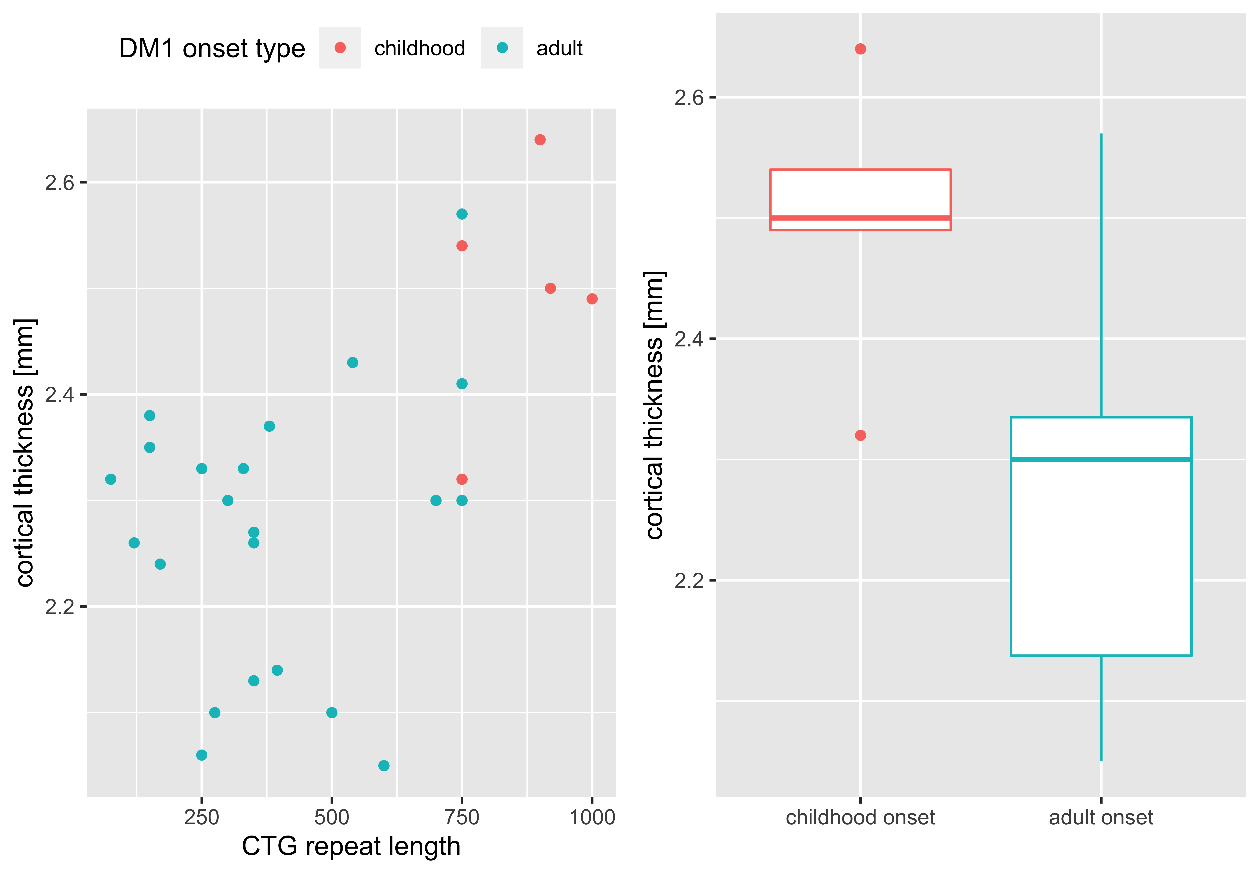

Supplement: Supplementary file 1 — Supplementary file1 (DOCX 2055 KB) [file 415_2024_12511_MOESM1_ESM.docx]
